# Endoscopic sutured purse-string resection: a novel technique for resection of large gastric subepithelial lesions

**DOI:** 10.1055/a-2299-1899

**Published:** 2024-04-24

**Authors:** Olaolu Olabintan, Theodoros Voulgaris, Homira Ayubi, Sri Thrumurthy, Amyn Haji, Bu'Hussain Hayee

**Affiliations:** 1 Gastroenterology & Advance Therapeutic Endoscopy Department, King’s College Hospital NHS Foundation Trust, London, United Kingdom; 2 Upper GI Surgery & Advance Therapeutic Endoscopy Department, King’s College Hospital NHS Foundation Trust, London, United Kingdom; 3 Colorectal Surgery & Advance Therapeutic Endoscopy Department, King’s College Hospital NHS Foundation Trust, London, United Kingdom

Subepithelial lesions (SELs) in the gastrointestinal (GI) tract are common and often necessitate removal, particularly when >20 mm 1 2 . The choice of endoscopic resection depends on various factors, including lesion characteristics, location, and evidence of deeper tissue involvement 2 3 . Challenges in achieving full-thickness resection have driven the development of innovative over-the-scope devices 4 . However, these devices are typically restricted to lesions <30 mm, and their size and rigidity often hinder passage beyond the pharynx. We present here a novel technique for accomplishing full-thickness resection of SELs, known as endoscopic sutured purse-string resection (ESPR). ESPR employs the Overstitch device (Apollo Endosurgery, Austin, Texas, USA), a well-established tool for placing full-thickness endoscopic sutures. It involves creating a purse-string configuration around the lesion ( Fig. 1 ) before resection, enabling the safe and complete removal of even larger lesions.

**Fig. 1:**
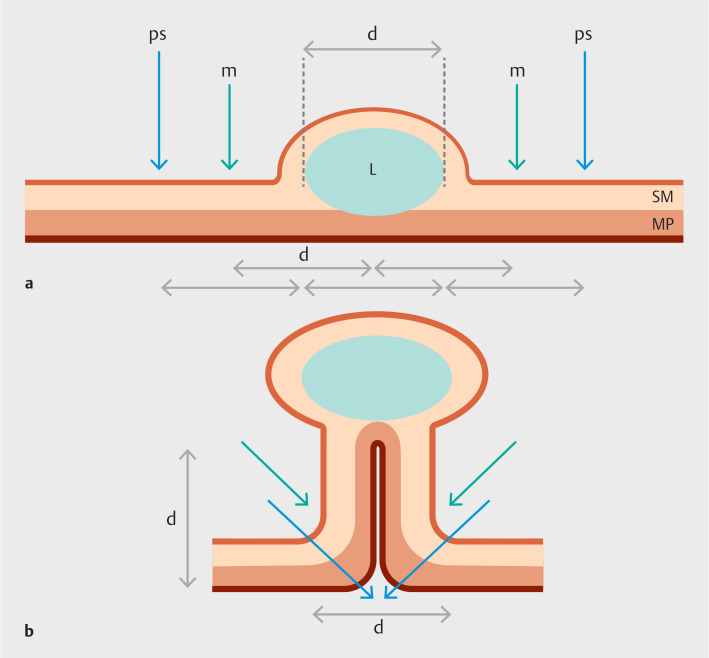
Schematic showing lesion marking and suture placement for endoscopic sutured purse-string resection. **a** Lesion (L) of diameter (d), which allows placement of mucosal diathermy marks (m) and purse-string sutures (ps). **b** Lesion after tightening of the purse string, with corresponding sites of mucosal marks and suture sites. Snare resection is conducted at or underneath the diathermy marks. SM, submucosal layer; MP, muscularis propria.

Patient selection followed European Society of Gastrointestinal Endoscopy guidelines, with a preference for lesions located on the greater curve of the stomach, primarily due to the easier access provided by the Overstitch device. Lesions underwent thorough characterization using endoscopic direct visualization, endoscopic ultrasound, and cross-sectional imaging (computed tomography), following a standardized protocol.

ESPR is conducted as follows ( Video 1 ): marking the boundaries of the lesion, applying an endoscopic purse string, tenting the lesion with forceps, and creating a pseudopolyp by tightening the purse string. Resection was carried out using a large snare, and the site underwent meticulous inspection for completeness before being oversewn with a Z-shaped suture ( Fig. 2 ). In the two cases where this technique was employed, no perioperative complications were encountered. Patients were discharged on the same day, and histological examination confirmed complete resection, including the muscularis propria and serosa layers, along with omental fat in one case.

**Fig. 2:**
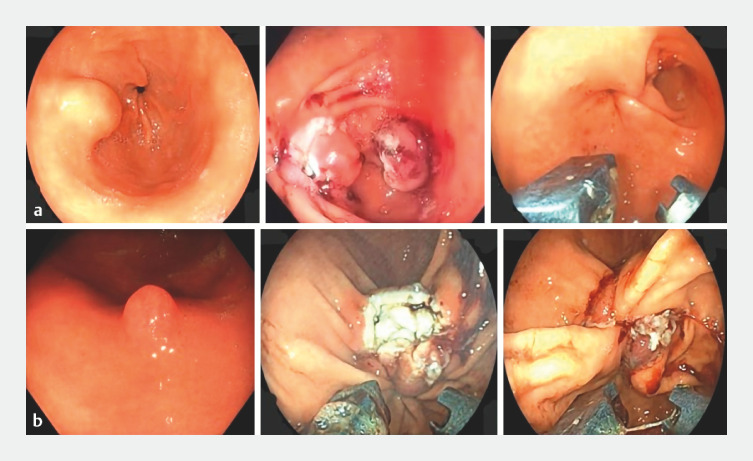
Image showing the two lesions ( **a** and **b** ) resected using the endoscopic sutured purse-string resection technique. **a1** 30-mm gastric antral subepithelial lesion. **b1** 15-mm greater curvature lesion. **a2** , **b2** Resected site showing complete resection. **a3** , **b3** Closure of the resected site with Z-shaped suture (4-suture point).

Endoscopic sutured purse-string resection for the removal of subepithelial lesions in the gastrointestinal tractVideo 1

This technique presents a safe and viable solution for resecting gastric SELs, effectively addressing the limitations of existing techniques. Its potential applicability extends beyond the stomach, offering a promising avenue for further exploration and adoption in the field of GI endoscopy.

Endoscopy_UCTN_Code_CCL_1AB_2AD_3AB

## Notes

Endoscopy E-Videos https://eref.thieme.de/e-videos
*E-Videos* is an open access online section of the journal *Endoscopy* , reporting on interesting cases and new techniques in gastroenterological endoscopy. All papers include a high-quality video and are published with a Creative Commons CC-BY license. Endoscopy E-Videos qualify for HINARI discounts and waivers and eligibility is automatically checked during the submission process. We grant 100% waivers to articles whose corresponding authors are based in Group A countries and 50% waivers to those who are based in Group B countries as classified by Research4Life (see: https://www.research4life.org/access/eligibility/ ). This section has its own submission website at https://mc.manuscriptcentral.com/e-videos .
